# Fostering nursing innovation to prevent and control antimicrobial resistance using approaches from the arts and humanities

**DOI:** 10.1177/1744987120914718

**Published:** 2020-05-03

**Authors:** Colin Macduff, Anne Marie Rafferty, Alison Prendiville, Kay Currie, Enrique Castro-Sanchez, Caroline King, Fernando Carvalho, Rick Iedema

**Affiliations:** Senior Research Fellow, School of Design, Glasgow School of Art, UK; Professor of Nursing Policy, Florence Nightingale Faculty of Nursing and Midwifery and Director of Academic Outreach, Academic Lead for Arts and Health, King’s College London, UK; Reader, Designing for Service, University of the Arts London, UK; Professor of Nursing and Associate Dean Research, School of Health & Life Sciences, Glasgow Caledonian University, UK; Consultant Nurse, Imperial College Healthcare NHS Trust, UK; Research Fellow, Safeguarding Health through Infection Prevention Research Group, School of Health & Life Sciences, Glasgow Caledonian University, UK; Research Assistant, RIPEN project, University of the Arts UK; Director, Centre for Team-based Practice & Learning in Health Care, Faculty of Life Sciences and Medicine, Kings College London, UK

**Keywords:** antimicrobial resistance, arts and humanities, imagination, infection prevention and control, innovation, nursing, visual methods

## Abstract

**Background:**

Efforts to address the complex global problem of antimicrobial resistance (AMR) highlight the need for imagination and innovation. However, nursing has not yet leveraged its potential to innovate to prevent AMR advancing.

**Aims:**

This paper focuses on the initial phase of an ongoing research and development study that seeks to foster nursing imagination and innovation by enhancing the meaningfulness of AMR for practising nurses and by facilitating their creative ideas.

**Methods:**

This aim is addressed through application of arts and humanities approaches, in particular the use of visualisation, co-design and historical methods, underpinned by the Design Council Double Diamond process model. The first phase with 20 UK participants explored how hospital and community-based nurses understand and respond to the priorities and consequences of AMR within their everyday working lives.

**Results:**

Nurses varied in their conceptualisations of AMR and in their depictions and explanations of its meaning and priority within everyday practices. Some saw infection prevention and control as bound up with AMR, whereas others differentiated in the context of specific work activities. Insights into related reasoning and practice tactics were also generated.

**Conclusions:**

The initial project phase provides a basis for fostering nursing innovation in this important field.

## Introduction

### Innovation, imagination and nursing practice

Before outlining one approach to fostering innovation in nursing, it would be useful to consider the concept of innovation itself. Within the context of health service delivery and organisation, Greenhalgh et al. (2004) define it as ‘a novel set of behaviours, routines and ways of working, which are directed at improving health outcomes, administrative efficiency, cost-effectiveness, or the user experience, and which are implemented by means of planned and coordinated action’. Through its clarity of emphasis on outcomes and related logical process, this definition has gained traction within healthcare literature.

Nevertheless, this only covers part of the story. It is necessary to ask how such innovation comes about, where it comes from and who is involved. One response to this is to associate innovation with invention and even genius. The famous maxim on the latter (often attributed to Thomas Edison), that ‘genius is 2% inspiration and 98% perspiration,’ typifies *first the spark, then the sweat* ideation. The limitation of this way of thinking, however, is that it tends to highlight isolated sparks rather than ongoing creativity in ways of thinking, seeing and improving practice.

Within this ambit we would argue that innovation in nursing may be more usefully considered as an ongoing response to a process of dialogue between practice as it is, and practice as it could or should be. At the heart of this dialogue is imagination in nursing. Stemming from the Latin verb ‘*imaginari*’, to ‘picture to oneself’ (Lexico (Oxford) English Dictionary 2020), ‘imagination’ thus places visualising centre-stage. Applied to innovation in nursing this entails the capacity to attend to (and engage in) ongoing dialogue between nursing as is and could be, and to either envisage and generate new ideas, or envisage adopting/adapting new knowledge and technologies in meaningful ways. As such, parts of this proactive envisaging go on in the *mind’s eye*, and Pearson et al.’s (2013) related psychology-based review suggests that such image generation is built using the scaffolding of previous related memorised perceptual information. Put simply this means that we tend to build from what we have experienced. While this is useful in terms of nursing as is, it highlights the need for stimulation to help envisage nursing as it could/should be. Accordingly, we would argue that the quality of nursing imagination and related innovation is likely to be enhanced by dialogue that incorporates knowledge and challenge from other disciplines within and beyond healthcare.These initial considerations underpin the approach in the present paper which focuses on the initial phase of an ongoing research and development study that seeks to foster nursing imagination and innovation by enhancing the meaningfulness of antimicrobial resistance (AMR) for practising nurses and by harnessing their creative ideas.

### The AMR challenge and nursing

AMR is now recognised as a global problem of high magnitude (World Health Organization, 2014). Within the UK, England’s previous Chief Medical Officer has warned of the ‘catastrophic threat’ that this rapid evolution of microbial resistance to antibiotics poses, given that the latter are one of the foundations of treatment (and, ironically, prevention) within modern healthcare (Davies, 2013). Overuse and inappropriate use of antibiotics are at the heart of the problem. Efforts to address this threat have accelerated since the millennium, with the UK being a leading advocate for change both internationally and through national action. A key part of this initiative involves the UK’s Research Councils (now constituted within UK Research and Innovation; UKRI) working jointly to advance inter-disciplinary programmes of work. This highlights the recognition that no one discipline alone can adequately tackle this complex issue and that innovation is not only required to create new antibiotics, but is also necessary to address the range of social, cultural, economic and behavioural issues that influence our decisions about whether or when and how to use antibiotics. Consequently, this presents an opportunity for nurses to positively contribute to this agenda, and this paper relates to one inter-disciplinary nursing-focused study funded by the Arts and Humanities Research Council (AHRC) as part of this UKRI initiative.

The need for nursing to harness its power to this end is pressing. Nurses constitute the largest professional healthcare workforce globally and, typically, nurses have numerous daily interactions with healthy and ill individuals, family members, community groups and other care professionals. Thus, they have many potential opportunities to enact antimicrobial stewardship (AMS) practices such as education to help lessen inappropriate demand for antibiotics or ensuring that these drugs are prescribed and administered optimally. However, to date, the profession has not yet leveraged its full potential to prevent AMR advancing or to countenance the consequences of failure. Survey research in the UK and beyond (e.g. Mostaghim et al., 2017; NHS Education for Scotland, 2014) indicates that nurses often struggle to substantively develop AMS practices within their roles, with low levels of understanding of AMS, time/workload constraints and ingrained habits and attitudes all cited as impeding factors. Within the UK, AMS has recently been incorporated into the Nursing and Midwifery Council (NMC) Standards of Proficiency (NMC, 2018), which provides a new impetus for improving practice. Looking beyond current practice, it seems that few individuals or organisations in nursing have fully envisaged the consequences for practice of a world with minimal or no effective antibiotics. Johnstone (2016) is one exception and she emphasises that such a situation will present practising nurses with profound everyday ethical issues such as applying ‘“altered standards of care” when the therapeutic expectations of antimicrobials can no longer be met’.

A number of AMR toolkits exist for staff (e.g. Public Health England, 2015) but, perhaps inevitably in a situation where there is pressing need, many of the AMR initiatives covering nursing so far have had a top-down tendency, telling staff what to do. Our situational analysis suggests that there is a relative lack of engagement with, and ownership of, this agenda, and that this is exacerbated by the invisibility and abstractness of the risk. As such there appears to be a problem around the meaningfulness of the espoused AMR agenda for enactment in practice. Based on our recent healthcare associated infection-focused work around visualisation of people within hospital practice (Iedema et al., 2013) and visualisation of pathogens in the mind’s eye within hospital practice (Macdonald et al., 2017; Macduff et al., 2013), we believe it likely that there is also underlying difficulty in imagining and visualising (a) everyday practices within settings (i.e. ecologies) that could help prevent AMR, and (b) the consequences of failing to adequately address AMR in terms of repercussions for the ecologies of practice within which nursing operates.

## Methodology

### The RIPEN study design

The Re-envisaging Infection Practice Ecologies in Nursing through Arts and Humanities Approaches (RIPEN) study seeks to address the issues outlined above by starting from where practising nurses are in their daily working lives. In doing so RIPEN uses, and reflexively evaluates, a combination of primarily visual-based qualitative methods drawn from design, art, history, health services, and policy research. The former three disciplines in particular are rooted in the arts and humanities and their approaches offer potential for different ways of thinking and seeing. Using these different windows and mirrors for the issue of AMR may have the potential to foster nursing imagination and innovation, and this rationale drives the study’s main question:

*How can relevant arts- and humanities-based approaches help nurses to re-envisage their infection control practice ecologies in response to antimicrobial resistance?*

In addressing this overarching question, four subsidiary questions are also explored, namely:

1. *How do groups of hospital- and community-based nurses understand and respond to the priorities and consequences of antimicrobial resistance (AMR) within the context of their everyday working lives?*
2. *How can co-design and visualisation-based approaches help these nurses to identify and construct sets of meaningful practices that optimise present prevention of AMR?*
3. *How can co-design, visualisation, history and other relevant arts and humanities approaches help nurses to re-imagine and re-envisage their infection control practice ecologies in a future with minimal or no effective antibiotics?*
4. *What priority issues and other questions does this initial enquiry raise, and how can these best inform policy and planning, education and further research?*

The study is designed around two practice ‘labs’, one in Glasgow with a small group of mainly hospital-based participants and one in London with a small group of mainly community-based participants. Over the course of a year the participants within each lab are attending four workshops that address each of the above subsidiary study questions in turn. In between the workshops participants can also participate in shared activities online through a virtual learning environment. Following these workshops, a final integrative ‘policy lab’ will be convened whereby the research team, a sub-group of participants from both lab sites, the study advisory group and an invited range of policy experts and arts and humanities academics will address the latter part of Question 4.

**Figure 1.**
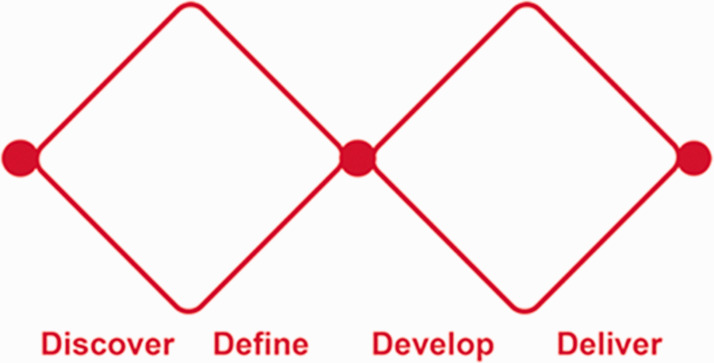
Framework of the Design Double Diamond process model.

The underpinning conceptual model is the four-stage Design Double Diamond process model (discover, define, develop, deliver; http://www.designcouncil.org.uk/news-opinion/design-process-what-double-diamond; see Figure 1), which is broadly concomitant with the progression of the four subsidiary questions and the sequence of workshops.

The discovery and development stages primarily involve processes of opening up (e.g. discovering nurses’ understandings and responses as explored through Question 1; and creative re-envisaging of practice ecologies in Question 3), while the defining and delivery stages aim more to focus down (e.g. identifying practices in Question 2; and final identification of priority issues in Question 4). As the initial discovery stage is now completed, this paper primarily focuses on methods and findings relating to Question 1.

The study’s design and conduct are also informed by participatory co-design thinking (Robert and Macdonald, 2017), building in some flexibility to evolve methods in response to participants’ ideas. Moreover, the research teams in Glasgow and London bring different skill sets to the project (Glasgow predominately nursing; London mainly design) and this has afforded scope for each lab to address the individual study questions from slightly different angles with some variation in methods. For the purposes of this paper, examples from the Glasgow lab are presented.

### Phase 1: Glasgow Workshop 1 methods

Table 1 summarises the main methods used during Glasgow Workshop 1 in terms of the design aim and related questions, activities, underpinning rationale, theoretical referents and analysis processes.

**Table 1.** Glasgow Workshop 1 methods and underpinnings.

Design aim	Key question/s	Activities	Rationale	Main theoretical influences/referents	Analysis Stage 1	Analysis Stage 2
Discover	How do participants picture AMR/aspects of AMR in their minds?	1. Each participant drawing AMR then pairs interviewing each other about the images, eliciting what is represented and why that content and form.	To creatively elicit conceptions that as yet are not well-documented in nursing literature and to explore the thinking behind these.	Sullivan’s Dimensions of Visualisation (Sullivan, 2005), viz. creative, descriptive, interpretive and explanatory aspects.	Grouping of images by orientation (e.g. processes or causes/effects?). Transcription of audio recordings. Charting of related key manifest visual and verbal (interview) content.	A pair of researchers explores this text and, where relevant, interprets any underlying meanings (latent content) in the light of related literature.
Discover	What and where are the main daily ‘touchpoints’ for participants in terms of AMR, IPC or both within their working lives? To what extent do participants differentiate AMR and IPC work, and what thinking informs this?	2. Each participant completes a storyboard picturing a typical day in their working life, including any contexts and activities related to AMR, IPC or both. Participants attach a green dot where activities seen as IPC-only, red where AMR-only, and yellow where IPC and AMR are concurrent. Numbering then indicates priority each activity is given within daily practice (1 = consistently very low priority, through to 5 = consistently very high priority). Participants can indicate if own priority-giving differs from others in team. Pairs interview each other about the storyboards in terms of general narrative and most meaningful frames. Eliciting what is going on, who is involved, and why is it happening in that way?	To explore understandings and responses to the priorities and consequences of AMR within the context of everyday work life, as these are not well-documented in nursing literature. To explore how AMR is defined and prioritised in relation to other work in a typical day as practitioners are being asked to be AMR stewards but the situated meaning and coherence of this are not well-understood. Through the process participants will gain reflective insights into their AMR practice and learn about and from others’. The research team will also learn more about the relative value of different methods.	Sullivan’s Dimensions of Visualisation, viz. creative (e.g. conceptualising), descriptive (e.g. mapping), interpretive (e.g. narrating) and explanatory (e.g. systematising) aspects. Normalisation process theory (NPT; http://www.normalizationprocess.org) – the first construct (coherence) of this implementation theory posits the importance of differentiation, specification and identification work to make sense of the relationship between new and existing practices. Mutual reflection on action (Schön, 1991)	Storyboards grouped initially by predominant framing context (i.e. hospital, community, multi-site/wide-ranging). Transcription of audio recordings. Focus on the key frames identified by participants – charting of related manifest visual and verbal (interview) content. Extraction of key quotes that provide rich insights (‘thick description’) re. practice heuristics, meanings, priorities and differentiations.	A pair of researchers explores this text and, where relevant, interprets any underlying meanings (latent content; Graneheim and Lundman, 2003) in the light of related literature. Collation of main trends in relation to the questions driving the workshop activities.

AMR = antimicrobial resistance; IPC = infection prevention and control; NPT = Normalisation Process Theory.

As Table 1 suggests, the workshop was designed to create a platform for future innovation by first creatively discovering more about how participants conceptualised AMR and its manifestations and meanings in everyday practice. This addresses a gap in nursing literature.

**Figure 2.**
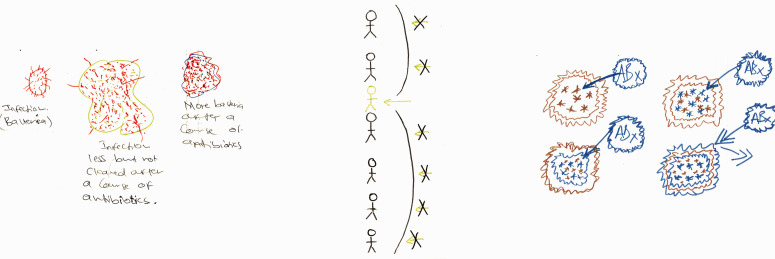
The micro lens: depicting processes of antibiotic resistance.

**Figure 3.**
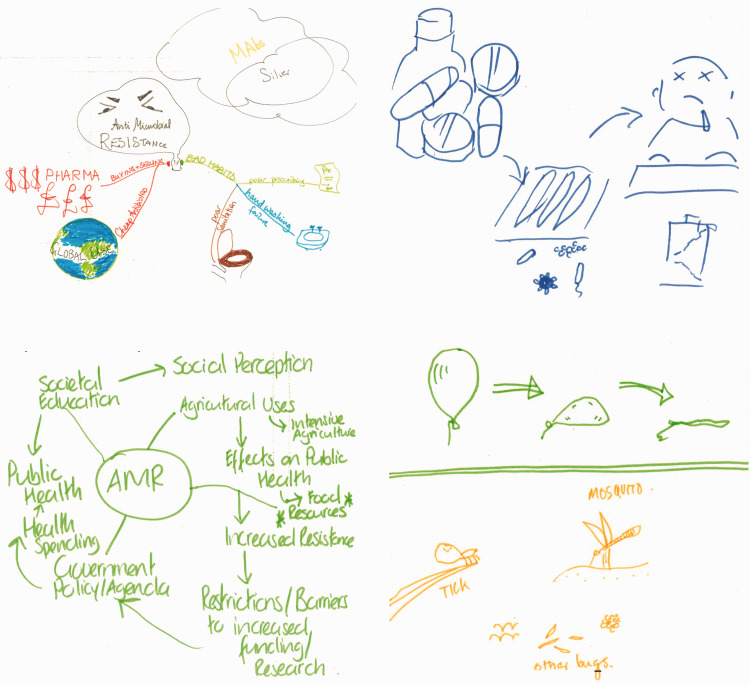
Victims, vectors and the bigger picture: drivers and consequences of antibiotic resistance.

**Figure 4.**
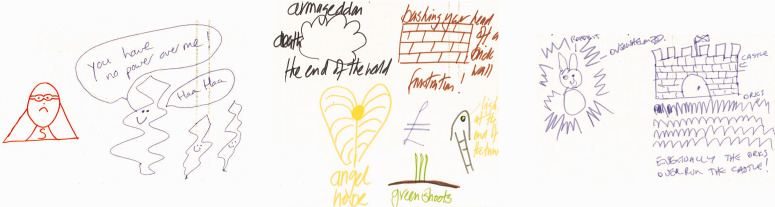
Metaphors and feelings: the conflictions of antibiotic resistance.

Activity 1 approached conceptualisation by asking individual participants to think of resistance to antibiotics and its causes and effects, then draw how they pictured AMR in their mind (see Figures 2–4). Subsequently participants interviewed each other in pairs to narrate what their images represented and to elicit reasons for the form and content depicted. This combination of images and elicitations engages with the creative, descriptive, interpretive and explanatory aspects bound up in visualisation, as highlighted in Sullivan’s Dimensions of Visualisation (Sullivan, 2005), recognising that images in themselves are not necessarily self-explanatory.

**Figure 5.**
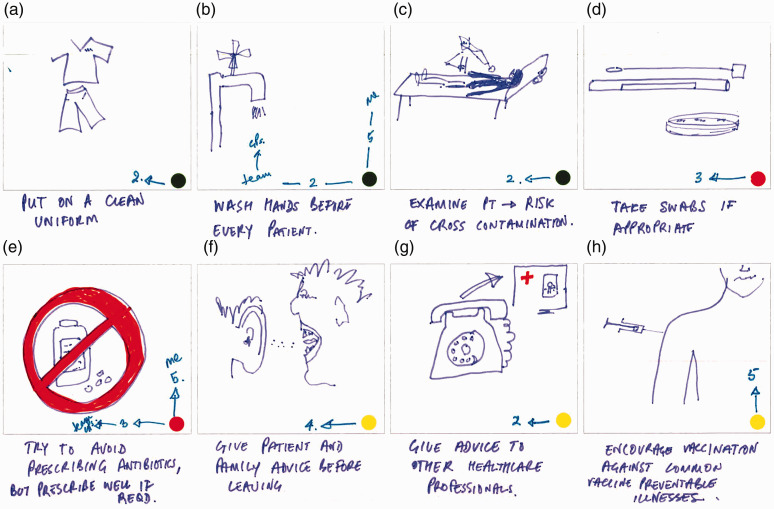
Day in working life: storyboard by an advanced practice nurse.

Activity 2 was designed primarily to elicit the main daily ‘touchpoints’ for participants in terms of AMR, infection prevention and control (IPC) or both within their working lives. Individuals completed an eight-frame storyboard (see Figures 5, 7 and 9 for examples of output), wherein they could indicate the nature of touchpoints and relative prioritisation of practices (see Table 1 for explanation of coloured dot system and numbering). Pairs then interviewed each other to elicit explanations of what was going on, where, with whom, how and why, which were audio-recorded. In the process they highlighted the most meaningful frames, any differentiation of AMR and IPC, and related individual and team priorities.

The Glasgow lab research team had a particular interest in understanding the extent to which nurses differentiate AMR work from IPC work. This specific angle stems from a gap in research literature and the idea that, if nurses are now being told to implement and normalise AMS, it is important first to understand (a) how they conceptualise AMR, (b) related AMR work, and (c) whether/how this differs from IPC work. The lens used is normalisation process theory (NPT; May and Finch, 2009), an implementation theory whose first construct (coherence) posits the importance of differentiation, specification and identification work to make sense of the relationship between new and existing practices. As such, it has particular relevance for learning about the coherence of AMR as a concept in practice as is and as could be.

Thus, visual methods were central to the workshop activities to help participants situate, visualise and explain their current thinking and practice relating to AMR in a meaningful way. In the process, implicit knowledge could be made explicit. In turn, this could facilitate mutual reflection on action (Schön, 1991) and promote imaginative thinking around practice improvements. Moreover, the visual methods in Workshop 1 were designed as a primer for subsequent workshops involving creative co-design of meaningful practices.

Such methods create a rich mix of visual, textual and audio data pertaining to AMR and nursing infection practice ecologies. Data analysis and synthesis of workshop outputs is driven by the main workshop questions, with extensive use of matrices that integrate the visual and textual material. These matrices facilitate consideration of manifest and latent content (Graneheim and Lundman, 2004) and researcher interpretation in the light of relevant theoretical perspectives. Reflexive evaluation of the challenges, strengths and any added value relating to the models, methods and materials used in the study is also being undertaken. This involves eliciting reflections at the end of workshops via a structured feedback form and group discussions. This data was collated for Workshop 1 and is drawn on in the findings below.

### Participants

For this exploratory study we wished to recruit around 10 nurses for each lab, with London focusing primarily on community nurses and Glasgow on hospital-based nurses. We sought to recruit mixed groups in terms of work settings and relative degrees of generalism, specialism and experience, as it was felt that this would enrich the nature and scope of dialogue. For the latter reason and to incorporate external perspectives we also sought participation from two patient/public representatives and two representatives from professions allied to nursing (AHPs) for each lab.

Study information was disseminated widely via the UK nursing press, professional and organisational networks, and volunteers responded by self-referring. The majority of participants were recruited this way and we had to decline participation from several specialist IPC nurses in order to ensure group balance. To help recruit more generalist community nursing participants in London we gained Integrated Research Approval System permission (19/HRA/0118) from two NHS trusts. The workshops took place on university premises and ethical permissions were obtained from the five collaborating universities.

The profile of the final groups of participants is summarised in Table 2. It was only possible to recruit one AHP (doctor) and one patient/public representative, both in Glasgow.

**Table 2.** Roles of the study participants.

London	Glasgow
District nurse team manager	Staff nurse (acute elderly ward)
Community mental health nurse	Staff nurse (various settings, mostly care homes)
Community nurse/DN student	Staff nurse (respiratory ward)
Rehabilitation nurse case manager	Advanced nurse practitioner (emergency care)
Community nurse (medical screening)	Advanced nurse practitioner (emergency care and public health role)
Community occupational health nurse	Community mental health nurse
Health adviser nurse	IPC nurse (hospital and rural community)
IPC consultant nurse (hospital group)	IPC consultant nurse (hospital group)
IPC nurse (Ministry of Defence)	IPC nurse (hospital group)
	Junior doctor
	Patient/public representative

DN = District Nurse; IPC = Infection prevention and control.

## Findings

This section presents findings contextualised through some analytic commentary.

### Activity 1: Picturing AMR

As the collated responses in Figures 2–4 show, drawn conceptions of AMR could broadly be summarised within three categories: 1) The micro lens 2) Victims, vectors and the bigger picture 3) Metaphors and feelings.

The depictions in Figure 2 are redolent of the view from microscope to Petri dish. Two portray a process of resistance at microcellular level, while the other lays out stickmen like tiny organisms to highlight the one case (green) where the drugs are working. Our previous work (Macduff et al., 2013) suggests that the microbiological lens is a powerful referent in nurses’ conceptions of pathogens. The link to practice was clear for one of the above depictors who talked of her work in a care home:

Because there was no culture done, there was nothing done, so we just prescribe any antibiotic and that’s not helping (Staff nurse, care home sector).

By contrast, the next grouping (Figure 3) focused either on the macro view (through factorial diagrams) or on victims (e.g. children) and vectors (e.g. tics, mosquitos). The macro depictions wrestle with the complexities of a global problem:

It’s a hybrid spider diagram. So what I have here is various offshoots of a nucleus of antimicrobial resistance. I think a major thing that can be traced to antimicrobial resistance is the use of intensive agriculture (Staff nurse; acute elderly ward).

There is a tension here between the merits of conceiving the bigger picture and the scope of any one health profession to act and innovate. This is amplified when viewing the Department of Health’s comprehensive but rather overwhelming factorial network depictions (www.gov.uk/government/publications/antimicrobial-resistance-amr-systems-map).

The final group of images tended to put personal feelings such as fear and frustration at the forefront, often expressed through metaphor:

As a professional working in that field I do feel that often I’m bashing my head off a brick wall trying to get the message across, and experience quite a lot of frustration, hence the brick wall (IPC consultant nurse, hospital group).

A staff nurse’s depiction of a disappointed antibiotic superhero facing victorious germ protagonists can be seen as part of visual genre with extensive historical roots (Stark and Stones, 2019) and it is notable that affect or ‘embodied emotional response’ is often used within visual mass media interventions to promote increased AMS amongst the public (Langdridge et al., 2018).

Taken together, the images and interpretations generated by participants indicate the nature and scope of conceptions that arise when nursing engages with AMR.

### Activity 2: Storyboarding

Storyboarding a typical working day was the central activity in Workshop 1. Examples from hospital, community and multi-site/wide-ranging role contexts are now presented along with analytic commentary.

**Figure 6.**
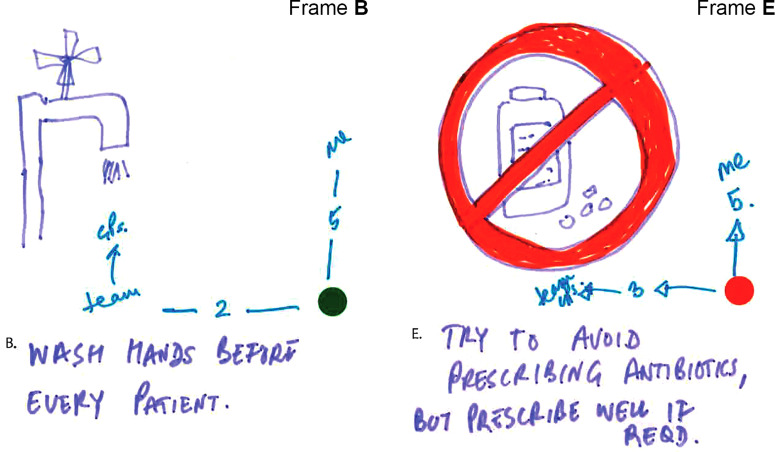
Close-up view of Frames B and E.

Figure 5 shows the storyboard from a very experienced advanced nurse practitioner who also has a public health role. In the initial frames routine generic clinical activities such as handwashing and examination are seen as IPC-only, while specific testing and prescription activities are AMR-only. The latter activities involving communication and vaccination are seen as having both IPC and AMR dimensions. Interestingly, distinctions are often made between the priority this nurse is giving to an activity and the priority that others in the team give it. This is seen clearly in Frame B (see close-up in Figure 6) in relation to handwashing but also in the AMR focal area of antibiotic prescribing (Frame E).

The related interview captures an example of a routine related to Frame B that is innovative in terms of serving more than one purpose:

I have a practice that whenever I bring a patient into a cubicle, I then wash my hands while I’m introducing myself and I partly do that because I think it’s polite, because the patient wants to know that you’re clean. So I could easily wash my hands before I go and get them, but I think, well if I do it in front of them, then it helps them build trust in me that here’s someone who is taking basic stuff seriously … but I think a lot of other people don’t do that (Advanced nurse practitioner, emergency care).

The interview also provides insight into another practice tactic relating to Frame E:

I mean the other thing that I try to do is try and play up the bad side of antibiotics … You’re saying ‘here’s something which has possibly got some benefit but has also got clear risks and they’re not just big world risks of antimicrobials, they’re risks to you that you’re not going to feel great if you start taking this antibiotic’. Then weigh up the balance then (Advanced nurse practitioner, emergency care).

Within the context of tackling AMR by reducing inappropriate prescription of antibiotics, this tactic can be understood as an example of ‘exnovation’, which Bekelis et al. (2017) characterise as scaling back a practice while not necessarily abandoning it.

**Figure 7.**
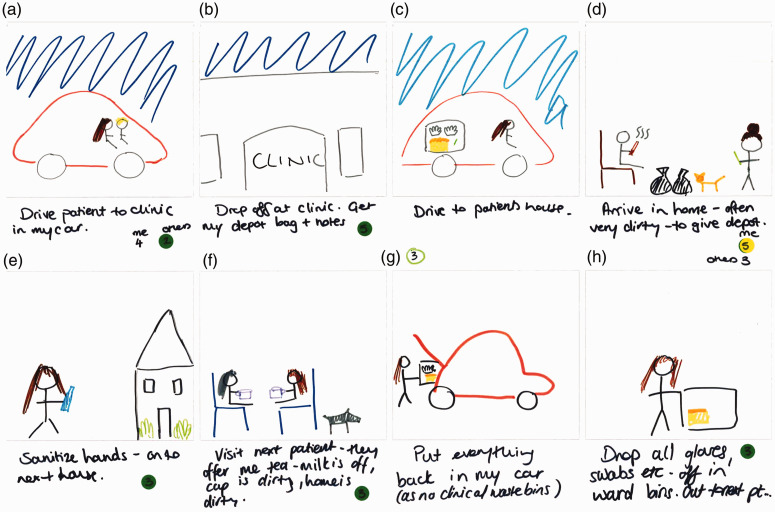
Day in working life: storyboard by a community mental health nurse.

A contrasting context, the world of a recently qualified community mental health nurse, is depicted in Figure 7. A key feature here is the flexibility of boundaries between the nurse and patient (e.g. sharing of car journey in Frame A) and related concerns about boundaries to contain potential microbiological risk in terms of cross infecting others (Frame G) and personal risk (Frame F). The following quotation illustrates the extensive emotional and practical labour related to IPC, which participants commonly talked about engaging in as they tried to make sense of and to navigate that risk, for themselves and for their patients.

Sometimes I park my car – there it is down the street. I need to sanitise my hands, put everything away, move onto the next house. The next house is going to have a whole new load of animals, and you know, I don’t mean germs in a rude way, but you know it is, everyone’s home is different and everyone has different things and if they want to give you a cup of tea and use their mug and so you are constantly being aware of potential for infection, I guess. And do your talking therapy work and then lots of other people in between (Mental health nurse, community).

While the vast majority of activities are seen in terms of IPC practice, administration of the depot injection (Frame D) involves a drug known for neutropenic effects and the nurse sees this as having related IPC and AMR considerations (although there is an indication on the storyboard that not all colleagues give this such priority).

Certain patients I go to see that are on clozapine or other high dose antipsychotics that do frequently get prescribed antibiotics, you do need to be aware of physical health (Mental health nurse, community).

The combination of visualisations and explanations here gives insights into how AMR is understood and contextualised within a broader topography of dynamic microbial and related IPC interactions involving humans, animals and diverse environments. This resonates with Hinchliffe et al.’s work (2013) on AMR and biosecurity, which highlights the limitations of focusing on rigid, defined boundaries (e.g. between home and hospital) and calls for ‘spatial imagination’, which better appreciates the fluid dynamics of ‘borderlands’ (e.g. multiple movements within and amongst homes, cars and hospitals).

The latter phenomenon is very much in evidence in the storyboard of an IPC consultant nurse, where role and setting diversity is the norm (Figure 9).

**Figure 8.**
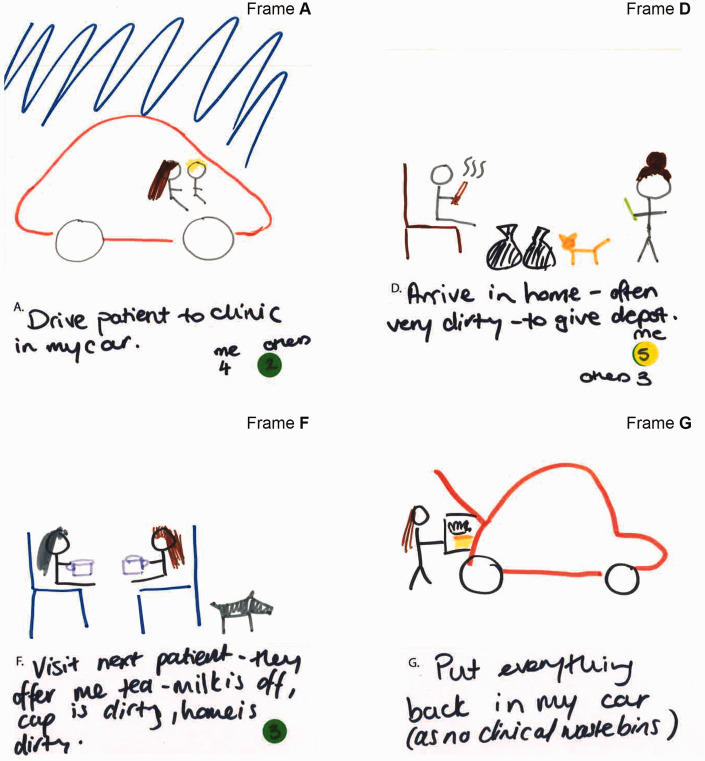
Close-up view of Frames A, D, F and G.

**Figure 9.**
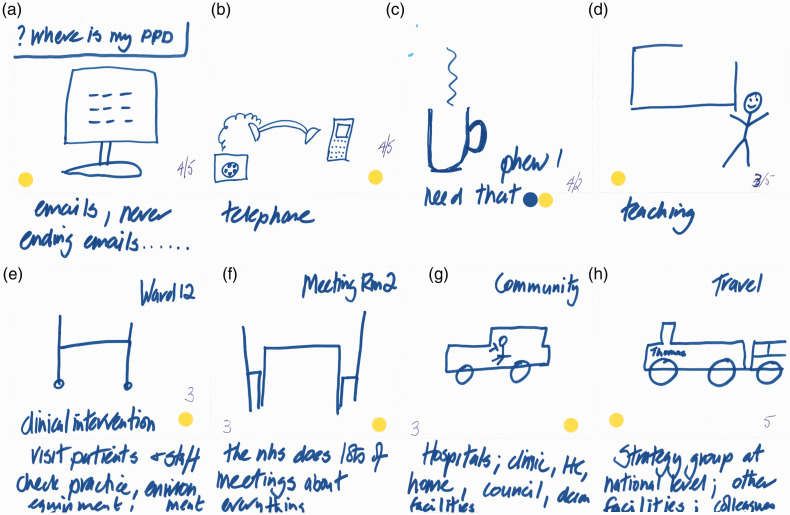
Day in working life: storyboard by an IPC nurse consultant.

This storyboard also highlights the tendency we found for IPC specialist nurses to see IPC as bound up with AMR, right through to the importance of staff self-hydration in counteracting AMR (see Frame C).

I’ve also put a yellow sticker which indicates that it’s about infection prevention and control and AMR, because for me hydration is vitally important for all of us … if we don’t look after ourselves, at the end of the day, we are also the patients. And we might end up being that little old lady who’s not got a good fluid intake habit developed over a lifetime and gets those unnecessary antibiotics (IPC consultant nurse, hospital group).

The same conception is evident in relation to hygiene:

So, for me, IPC and AMR are really intertwined in ways that I think a lot of people don’t really see. So for example in the previous exercise one of the participants had a bucket and a mop to show a cleaner and chose to say that that was just an infection prevention and control intervention, but actually it does remain an AMR intervention because by cleaning you are keeping down the bioburden in that room and therefore the potential resistance, microbial resistance, to each other. (IPC consultant nurse, hospital group)

The latter passage also shows the awareness amongst participants of differences in the way they are conceiving and acting in relation to AMR, and the mutual learning that took place within the workshops.

### Participant evaluations

Comments from participants (via the anonymous feedback form) were all very positive in regard to the meaningfulness and value of the activities, with the storyboarding being seen as particularly useful:

The picture boards – allowing me to fully understand different roles and the implication of infection and AMR.

This reflected a very strong generic theme in feedback, namely learning about the topic in detail and in overview through individual and group reflections:

Great to be able to take time out to think about the bigger picture, and put each person's role into overall context*.*

Nevertheless, the diversity of roles, contexts and experience also highlighted challenges:

Interesting to observe the differences of understanding of AMR, antibiotics and antimicrobials in a group of health professionals – so much work to be done!

The emphasis on visual aspects in the methods was very well received with many participants being agreeably surprised at their ability to successfully convey their thinking in these forms. Some adjustment was often necessary though:

It took me a while to change my way of thinking from text to picture based.

## Discussion

The workshop sought to use imaginative, generative methods to gain initial understandings of nurses’ conceptions of AMR and what it means to them within the context of their working lives, that is, nursing as is. Findings suggest a wide range in conceptions of AMR and in how it is seen (or, perhaps more accurately, imagined) to be manifest in practice. For some staff (e.g. IPC specialists) there often seemed no differentiation between IPC and AMR in the context of daily work; one was seen as very much bound up with the other. Other staff clearly differentiated the two in the context of specific work activities. In addition to perceived variations in conceptions of AMR within teams and across contexts, variations in the levels of priority being given to it were often highlighted. Seen through the lens of NPT (May and Finch, 2009), these findings suggest some difficulties around the coherence and specification of AMR as a focus for implementing new practices. This suggests a need for more in-depth research.

At one level such findings may seem unremarkable – variation is a common feature of health services. However, as reflected in the integrative review by Monsees et al. (2017), there is a dearth of qualitative research into nursing and AMR, particularly in terms of any studies that start from contextualised practice, include community nursing and have an ambit wider than solely antibiotics and prescribing. The present study uses novel methods to generate distinctive insights into what AMR means for nurses in practice, and highlights why trying to change practice through guidelines and top-down invocation is likely to be insufficient to leverage nursing’s potential in this field. These insights, and the ways of seeing that the visual-based methods seek to foster, also create a dynamic for subsequent imagining of nursing working practices as could be. These aspects are being addressed through subsequent workshops.

By helping to capture implicit conceptions, contexts, tactics, commonalities and differences in relation to AMR and IPC in the world of practice, these methods are useful in illuminating how people view and respond to an essentially invisible concept such as AMR. They are also productive in illuminating why they see it in a particular way. When the IPC consultant nurse argues that re-hydrating through a mid-morning drink (see Figure 9) is also about helping to prevent AMR, a chain of reasoning is apparent that telescopes AMR from the seemingly distal and abstract towards the present and proximal. Within this context the methods have also helped to highlight perceptions that the microbial world respects neither social boundaries between professional and patient (see Figure 7 and the cup of tea dilemma), nor distinctions between the professional’s working and personal life.

The individual methods being used in the RIPEN study are not in themselves new. For instance, they share elements with experience-based co-design and participatory visualisation approaches. Rather, where some claim to innovation may be made is in the inter-disciplinary combinations of methods outlined above, and in their application to this particular context (in the relative absence of in-depth qualitative approaches and amidst the domination of the microbiological lens). Part of the value of an exploratory study such as this lies in trying and testing ideas and new combinations of methods in context.

Indeed, as this paper goes to press and the workshop phases complete, more evidence of cumulative impacts for participants is emerging:

I’ve really enjoyed the creativity and the lateral thinking, and how to put science and art, joining the two together. They are seen as different but they can be very collaborative and informative. It’s been revelatory and enlightening in a lot of ways.

Working with the arts based method really makes you think about what you are doing and probably challenges, maybe people like me who probably think about things in a kind of probably one-dimensional method whereas this has made me think on a much wider scale and really made me think about what we are going to do in future.

Experiences of different practitioners very useful and allowed me to introduce new ways of working in regards to my practices in infection control areas.

## Conclusions

In concluding, we believe that the initial phase of this study has yielded some novel insights into nurses’ thinking and practice in relation to AMR. These understandings provide a platform for the ongoing study to foster innovation in nursing practice, and in doing so to also encourage related development of arts and humanities approaches in research and education in this field. This study seeks to show that such approaches are relevant to nurses’ work in addressing AMR within a range of contexts; for nursing is diverse and lives across homes, hospitals and hinterlands, through hands and feet, and in hearts and heads where imagination and innovation can thrive given nourishment. As Rafferty urges in relation to these many facets of nursing, ‘let’s not get boxed in’ (Scott, 2019).

## Key points for policy, practice and/or research

- Nursing has not leveraged its full potential to address antimicrobial resistance and it needs to facilitate nurses’ innovation in this area of practice
- By using arts and humanities approaches this research and development project aims to address these needs
- The initial phase used primarily visual methods to explore how hospital and community nurses understand and respond to AMR
- Nurses varied in their conceptualisations of AMR and in their practice responses to it
- The potential for further co-design work to harness nurses’ creativity and imagination is highlighted

## References

[bibr1-1744987120914718] Bekelis K Skinner J Gottlieb D, et al.(2017) De-adoption and exnovation in the use of carotid revascularization: retrospective cohort study. BMJ 359: j4695. 29074624 10.1136/bmj.j4695 PMC5656975

[bibr2-1744987120914718] Davies SC (2013) Annual report of the Chief Medical Officer. Volume Two, 2011. Infections and the rise of antimicrobial resistance, London: Department of Health. 10.1016/S0140-6736(13)60604-2 23489756

[bibr3-1744987120914718] Graneheim U Lundman B (2004) Qualitative content analysis in nursing research: concepts, procedures and measures to achieve trustworthiness. Nurse Education Today 24(2): 105–112. 14769454 10.1016/j.nedt.2003.10.001

[bibr4-1744987120914718] Greenhalgh T Robert G Macfarlane F, et al.(2004) Diffusion of innovations in service organisations. The Milbank Quarterly 82(4): 581–629. 15595944 10.1111/j.0887-378X.2004.00325.x PMC2690184

[bibr5-1744987120914718] Hinchliffe S Allen J Lavau S, et al.(2013) Biosecurity and the topologies of infected life: from borderlines to borderlands. Transactions of the Institute of British Geographers 38(4): 531–543.

[bibr6-1744987120914718] Iedema R Mesman J Carroll K (2013) Visualising Healthcare Practice Improvement: Innovation from Within, London: Radcliffe.

[bibr7-1744987120914718] Johnstone M (2016) Editorial: the moral significance of antimicrobial resistance and the rise of ‘apocalyptic superbugs’. Journal of Clinical Nursing 25(15–16): 2079–2082. 27278288 10.1111/jocn.13350

[bibr8-1744987120914718] Langdridge D Davis ML Gozdzielewska L, et al.(2018) A visual affective analysis of mass media interventions to increase antimicrobial stewardship amongst the public. British Journal of Health Psychology 24(1): 66–87. 30221433 10.1111/bjhp.12339 PMC6585774

[bibr9a-1744987120914718] Lexico (Oxford) English Dictionary (2020). https://www.lexico.com/definition/imagination (accessed 19 March 2020).

[bibr9-1744987120914718] Macdonald A Macduff C Loudon D, et al.(2017) Evaluation of a visual tool co-developed for training hospital staff on the prevention and control of the spread of healthcare associated infections. Infection, Disease and Health 22(3): 105–116. 10.1016/j.idh.2017.06.002 31862086

[bibr10-1744987120914718] Macduff C Wood F Hackett C, et al.(2013) Visualising the invisible: applying an arts-based methodology to explore how healthcare workers and patient representatives envisage pathogens in the context of healthcare associated Infections. Arts and Health 6(2): 117–131.

[bibr11-1744987120914718] May C Finch T (2009) Implementing, embedding, and integrating practices: an outline of normalization process theory. Sociology 43(3): 535–554.

[bibr12-1744987120914718] Monsees E Goldman J Popejoy L (2017) Staff nurses as antimicrobial stewards: an integrative literature review. American Journal of Infection Control 45(8): 917–922. 28768593 10.1016/j.ajic.2017.03.009

[bibr13-1744987120914718] Mostaghim M Snelling T McMullan B, et al.(2017) Nurses are underutilised in antimicrobial stewardship – results of a multisite survey in paediatric and adult hospitals. Infection, Disease and Health 22(2): 57–64.

[bibr14-1744987120914718] NHS Education for Scotland (2014) *Exploring the role of nurses and midwives in antimicrobial stewardship*. Available at: http://www.nes.scot.nhs.uk/education-and-training/by-theme-initiative/healthcare-associated-infections/educational-programmes/antimicrobial-resistance-and-stewardship.aspx (accessed 19 March 2020).

[bibr15-1744987120914718] Nursing and Midwifery Council (2018) Future Nurse: Standards of Proficiency for Registered Nurses, London: NMC. 10.1016/j.nedt.2024.106284 38870582

[bibr16-1744987120914718] Pearson D Deeprose C Wallace-Hadrill S, et al.(2013) Assessing mental imagery in clinical psychology: a review of imagery measures and a guiding framework. Clinical Psychology Review 33(1): 1–23. 23123567 10.1016/j.cpr.2012.09.001 PMC3545187

[bibr17-1744987120914718] Public Health England (2015) *Start smart* – *then focus: antimicrobial stewardship toolkit for English hospitals*. Available at: https://www.gov.uk/government/publications/antimicrobial-stewardship-start-smart-then-focus (accessed 19 March 2020).

[bibr18-1744987120914718] Robert G Macdonald A (2017) Co-design, organisational creativity and quality improvement in the healthcare sector: ‘designerly’ or ‘design-like’? In: Sangiorgi D Prendiville A (eds) Designing for Service. Key Issues and New Directions, London: Bloomsbury Academic Chapter 9, pp 118–130.

[bibr20-1744987120914718] Schön D (1991) The Reflective Practitioner, Aldershot: Ashgate Publishing Ltd.

[bibr21-1744987120914718] Scott K (2019) Meet our new RCN President, *RCN Bulletin*, 3 January. Available at: https://www.rcn.org.uk/magazines/bulletin/2019/january/meet-your-new-rcn-president (accessed 19 March 2020).

[bibr22-1744987120914718] Stark J Stones C (2019) Constructing representations of germs in the twentieth century. Cultural and Social History 16(3): 287–314.

[bibr23-1744987120914718] Sullivan G (2005) Art Practice as Research: Inquiry in the Visual Arts, Thousand Oaks: Sage.

[bibr24-1744987120914718] World Health Organization (2014) *Antimicrobial resistance: global report on surveillance 2014.* Available at: https://www.who.int/drugresistance/documents/surveillancereport/en (accessed 19 March 2020).

